# Radiographic reporting in adolescent idiopathic scoliosis: Is there a discrepancy comparing radiologists’ reports and surgeons’ assessments?

**DOI:** 10.1093/pch/pxae113

**Published:** 2025-02-25

**Authors:** Kara Sidhu, Marina Rosa Filezio, Vishwajeet Singh, Manjot Birk, David Parsons

**Affiliations:** Cumming School of Medicine, University of Calgary, Calgary, Alberta, Canada; Department of Clinical Neuroscience, University of Calgary, Calgary, Alberta, Canada; Cumming School of Medicine, University of Calgary, Calgary, Alberta, Canada; Department of Surgery, University of Calgary, Calgary, Alberta, Canada; Department of Surgery, University of Calgary, Calgary, Alberta, Canada; Cumming School of Medicine, University of Calgary, Calgary, Alberta, Canada; Department of Surgery, University of Calgary, Calgary, Alberta, Canada; Cumming School of Medicine, University of Calgary, Calgary, Alberta, Canada; Department of Surgery, University of Calgary, Calgary, Alberta, Canada

**Keywords:** Adolescent idiopathic scoliosis, Bracing, Cobb angle, Risser stage, Radiographic assessment, Inter-rater reliability, Intra-rater reliability, Quality improvement

## Abstract

**Objectives:**

Cobb angle is a standard method for quantification of scoliosis in adolescent idiopathic scoliosis to guide treatment decisions. Precise and timely curve detection can ensure early referrals, amenable for bracing. Radiology reports serve as a guiding tool for family physicians to expedite specialist referrals. Therefore, accurate and reliable measurement of Cobb angle at the community level is crucial. This retrospective study investigated the agreement in Cobb angle measurement between radiologists and spine surgeons.

**Methods:**

Eighty radiographic reports (Cobb angle, Risser stage, and end vertebrae selection) completed by radiologists and spine surgeons were compared. To assess interrater reliability, interclass correlation coefficients (ICC) with 95% confidence intervals (CIs) were computed. ICC < 0.70, 0.70 to 0.79, 0.80 to 89, and 0.9 to 0.99 were considered poor, fair, good, and excellent reliability, respectively. All radiographs were assessed for quality.

**Results:**

The agreement between spine surgeons and radiologists was poor (ICC = 0.65, 95% CI: 0.13 to 0.97). The agreement between spine surgeons and community radiologists was poor (ICC = 0.45, 95% CI: 0.17 to 0.66). Risser stage was not reported in 56 of the 80 reports. ICC between spine surgeons and radiologists for the Risser stage was poor (ICC = 0.625, 95% CI: 0.325 to 0.794). For end vertebrae identification, there was absolute agreement of end vertebrae identification in 23 of the 80 scans.

**Conclusions:**

This study demonstrated a significant disagreement in scoliosis measurement between radiologists and spine surgeons, which significantly impacts appropriateness of referrals. Methods to improve triaging using allied health professional (i.e., nurse practitioners) may help ensure that patients presenting with scoliosis are referred in a timely manner.

## INTRODUCTION

Scoliosis is a 3D spinal deformity involving one or more spinal curves with lateral deviation and axial rotation of the vertebrae (1). Adolescent idiopathic scoliosis (AIS) is the most common form of scoliosis in the pediatric population during puberty (1). Cobb angle is a standardised measurement which quantifies the degree of scoliosis and guides clinical decision-making in treating AIS, recognised by the Scoliosis Research Society (2). Cobb angle measurements are also used for surgical planning and evaluating the effectiveness of treatment. In our practice, primary care physicians rely on radiology reports to follow guidelines provided by specialists to facilitate priority setting of referrals at the tertiary care level. Accurate clinical and radiographic assessments at the primary care level are key to ensure timely referral to tertiary care, as well as appropriate triaging by tertiary centres which impacts wait times.

Treatment options for AIS include observation, bracing, or corrective surgery based on curve magnitude and skeletal maturity (determined by Risser stage). Results of the BrAIST study demonstrated bracing in children with a Cobb angle of 20º to 40º could lead to avoidance of invasive surgery in 72% of patients (3). This exemplifies the importance of precise Cobb angle measurements in ensuring that patients are referred to tertiary care, while they are still candidates for bracing, a time-sensitive, evidence-based intervention while preventing surgery and the complications associated with it. Furthermore, accurate Cobb angle measurements prevent inappropriate referrals that can often result in longer wait times for patients to tertiary care.

The Scoliosis Research Society has developed the following treatment recommendations: (1) suitable for management at the primary care level (Cobb angle 11°C to 24°C), (2) suitable for nonoperative management (i.e., brace treatment; Cobb angle 25°C to 40°C), or (3) suitable for operative treatment (or a late referral; Cobb angle > 40°C) (4).

Despite the importance of Cobb angle measurements in decision-making, its accuracy and reliability between clinicians in primary and tertiary care centres have not yet been investigated. Evaluation of intra- and inter-rater reliability of Cobb angle measurements has been demonstrated exclusively in tertiary care clinicians, specifically among orthopaedic surgeons. Similar reliability studies have yet to be done among radiologists, who are often responsible for measurement reporting in the primary care setting. Excellent intra- and inter-rater reliability (interclass correlation coefficient, ICC, above 0.90) has been reported among orthopaedic surgeons (5–9). End vertebrae identification of scoliosis curves was reported as the primary source of variability in Cobb angle measurements. Studies where end-vertebrae were predefined demonstrated better intra- and inter-rater reliability in comparison to studies where they were not predefined (10). Furthermore, technological advancements such as computerised digital radiogram have shown improved reliability and reduced variation in measurements compared with manual methods (11–13).

Radiologists play an essential role in scoliosis screening and tertiary care referrals. They are responsible for measuring Cobb angles and reporting them to primary care physicians, who are then responsible for making decisions on referral to tertiary care. Therefore, not only is the accuracy and reliability of Cobb angle measurement essential, but also the agreement of this measurement between radiologists and treating spine surgeons. High agreement between these two groups would ensure that patients have timely access to tertiary care, while they remain candidates for noninvasive therapy. The purpose of this study is to improve screening at the primary care level by investigating the agreement of the Cobb angle measurement between community radiologists, paediatric radiologists, and treating paediatric spine surgeons.

## METHODS

This study was approved by the University of Calgary’s Research Ethics Board (REB21-0389).

A retrospective review was conducted to evaluate spinal radiographs and reports of AIS patients from the initial referrals to a tertiary care centre for treatment. Eighty patients were randomly selected from our database. Inclusion criteria for radiographs included: (1) ages 10 to 18 years old, (2) referral made to Alberta Children’s Hospital between 2013 and 2018, and (3) clinical diagnosis of AIS. Exclusion criteria included: (1) nonidiopathic scoliosis (i.e., neuromuscular, congenital), (2) other musculoskeletal conditions (i.e., idiopathic juvenile arthritis, hypophosphatasia, and osteogenesis imperfecta), and (3) previous bracing or manipulation treatment by a chiropractor or physiotherapist. Each standardised scoliosis radiograph was reviewed in anterior-posterior (or posterior-anterior) plane. The end vertebrae, as well as curve magnitudes and skeletal maturity were recorded for each patient using the Cobb angle and Risser grade, respectively. Two independent fellowship-trained spine surgeons (*MRF* and *VS*), blinded to the reports, recorded all measurements on the same set of radiographs.

Similarly, Cobb angle, Risser stage, and end vertebrae were recorded from the radiographic reports obtained at various community imaging centres or tertiary care centres. Radiographs captured in the community were interpreted by radiologists in the community and radiographs captured at the tertiary care centre were interpreted by radiologists who have received specialised training in pediatric imaging.

An institutional picture-archiving computer system (IMPAX© Solution, AFGA HealthCare©, Mortsel, Belgium), available in the entire province, was used for the interpretation of all radiographs. Imaging studies were also reviewed for completeness; an anterior-posterior or posterior-anterior radiograph of the whole spine standing, including shoulders and pelvises, was considered standard and complete. Separate thoracic or lumbar radiographs were marked as incomplete, and repeat imaging was performed. Additionally, a report was complete if it commented on end vertebrae of the curve, Cobb angle of the structural curve, and Risser stage. An agreement between major curves and end vertebrae was compared for end vertebrae identification.

Reliability was defined as the repeatability of measurements and was determined based on assessing the agreement of different raters viewing the same material. Greater reliability indicated less variation of the same measurement. To compute reliability between raters in our study, ICCs with 95% confidence intervals (CIs) were computed. ICCs are based on two-way random-effects model utilising absolute agreement. ICC < 0.70, 0.70 to 0.79, 0.80 to 89, and 0.9 to 0.99 were considered poor, fair, good, and excellent reliability, respectively. For intra-rater reliability, comparisons were performed within the same speciality: (1) pediatric and community radiologists and (2) pediatric spine fellows. For inter-rater reliability, comparisons were preformed between the following groups: (1) all radiologists and pediatric spine fellows, (2) pediatric radiologists and pediatric spine fellows, and (3) community radiologists and pediatric spine fellows. These comparisons were performed for Cobb angle and the Risser stage.

## RESULTS

The mean age of participants was 13.3 ± 1.5 years (range 10 to 17), of which 62 (78%) were female. Forty scans were captured in the community, and 40 scans were captured at the tertiary centre, for a total of 80 scans. Of the 80 radiographs, 45 scans (56.3%) were complete according to the standardised criteria; however, 35 scans (43.8%) were not and repeat imaging was required at the time of their consultation visit. Forty-six of the 80 scan reports (57.5%) did not include comments on the Risser stage, and 14 (17.5%) reports did not comment on the end vertebra, and two reports did not comment on the Cobb angle.

**Figure 1.**
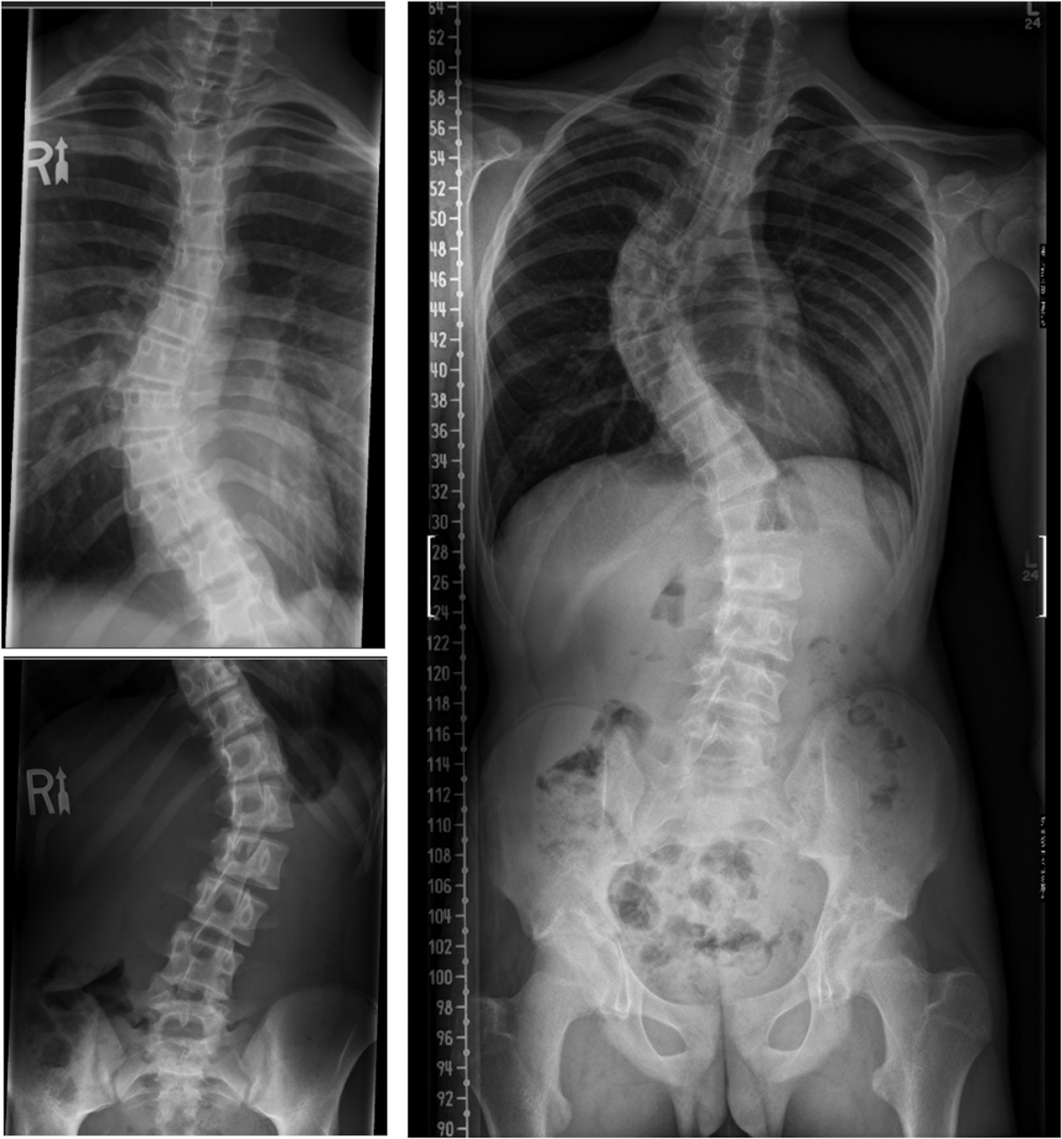
Radiographs of adolescent idiopathic scoliosis in 12-year-old patient. (Left) Radiographs performed in a community centre. Cobb angle of a single curve reported as 19°C. No Risser stage reported. (Right) Repeat radiograph during consultation for same patient. Two curves identified: thoracic and lumbar curve measuring 46°C and 59°C, respectively. Risser stage of 3 reported.

Intra-rater reliability between pediatric spine surgeons for Cobb angle measurements was excellent (ICC = 0.958, 95% CI: 0.936 to 0.973; Table 1). Inter-rater reliability between radiologists and pediatric spine surgeons for Cobb angle was fair (ICC = 0.652, 95% CI: 0.486 to 0.769). Inter-rater reliability between tertiary-care/pediatric radiologists and pediatric spine surgery fellows for Cobb angle was good (ICC = 0.874, 95% CI: 0.455 to 0.955). Inter-rater reliability between community radiologists and pediatric spine surgeons for Cobb angle was poor (ICC = 0.448, 95% CI: 0.165 to 0.664). Figure 1 demonstrates a case example of a community radiologist’s spinal measurements to a spine surgeon’s report of the same spinal radiograph.

**Table 1.** Interclass correlation coefficients between spine surgeons and radiologists

	Cobb angle	Risser stage
Spine Surgeons	0.958 (95% CI: 0.936 to 0.973)	0.847 (95% CI: 0.745 to 0.907)
Spine Surgeons and Radiologists	0.652 (95% CI: 0.486 to 0.769)	0.625 (95% CI: 0.325 to 0.794)
Spine Surgeons and Pediatric Radiologists	0.874 (95% CI: 0.455 to 0.955)	0.704 (95% CI: 0.704 to 0.826)
Spine Surgeons and Community Radiologists	0.448 (95% CI: 0.165 to 0.664)	0.277 (95% CI: 0.564 to 0.851)

Abbreviation: CI, confidence interval.

Intra-rater reliability between pediatric spine surgeons for Risser stage was good (ICC = 0.847, 95% CI: 0.745 to 0.907). Risser stage’s inter-rater reliability between radiologists and pediatric spine surgeons was poor (ICC = 0.625, 95% CI: 0.325 to 0.794). Inter-rater reliability between tertiary-care/pediatric radiologists and pediatric spine surgeons for Risser stage was fair (ICC = 0.704, 95% CI: 0.704 to 0.826). Inter-rater reliability between community radiologists and pediatric spine surgeons for Risser was poor (ICC = 0.277, 95% CI: 0.564 to 0.851).

For end vertebrae identification, there was absolute agreement of end vertebrae identification in 23 of the 80 scans (29%). Sixteen of these reports (61%) were completed by a pediatric radiologists, and the remainder were from the community. Three of the 80 scans only identified a single curve, while the spinal surgeons identified two curve angles. One of these three cases did not identify the major curve, rather the minor curve, only.

## DISCUSSION

AIS is amenable to noninvasive bracing therapy with early curve detection. Timely diagnosis and intervention for managing AIS relies on accurate measurement of the Cobb angle. The findings from this study report significant disagreement in Cobb angle measurements between radiologists and pediatric spine surgeons. The discrepancies were more remarkable between community radiologists and treating spine surgeons. This study also demonstrated that Risser staging had significant disagreement between radiologists and pediatric spine surgeons, regardless of the location of radiographic interpretation. Moreover, half of the reports coming from the community was not performed as per the standard guidelines. Findings from this study suggest that this discrepancy might negatively impact timely and appropriate referrals of AIS patients to tertiary care centres.

Literature examining the reliability of Cobb angle measurements in AIS has been limited to spine surgeons, and there has been no investigation to date comparing measurements between radiologists and spine surgeons. Specifically, there have been no comparisons performed between radiographs captured and analysed in the community versus a tertiary care centre. At the tertiary level, excellent agreement of Cobb angle measurements has been reported, including junior spine surgeons (5–9). Findings from our study support previous literature when calculating reliability between spine surgeons.

Variation of 5° or greater is considered clinically significant. Major sources of variation include the method of capturing the radiographic images and the selection of the end vertebrae (8,14). In this study, over one-third of the scans were not completed according to the standard guidelines (weight-bearing, full spinal length). Majority of the scans, primarily performed in the community, captured the thoracic and lumbar spine separately. This required children to undergo repeat spinal imaging in the specialty clinic at the time of their appointment. Regarding end vertebrae selection, there was a discrepancy in selection in 23 of the 80 scans. Moreover, there were three cases where only one spinal curve was identified when there were two to measure. This resulted in a single case where the incorrect curve was identified as a major curve.

Though the reliability of Cobb angle measurements was the primary variable of interest, Risser stage measurements were also compared. To our surprise, there was significantly poor reliability of Risser stage reporting between spine surgeons and radiologists. Furthermore, over half of the reports did not comment on the Risser stage. Risser stage provides a grade on skeletal maturity based on the level of ossification and fusion of the iliac crest apophyses. In conjunction with the Cobb angle, Risser stage helps to guide the treatment course, specifically for bracing therapy eligibility. For example, children who are of Risser stage four or greater and have a Cobb angle of greater than 40°C do not benefit from bracing therapy as they have reached spinal maturity and will likely require corrective spinal surgery. Unfortunately, previous studies have solely investigated the agreeability of the Cobb angle without comment on Risser stage.

Cobb angle measurements are also utilised in other clinical populations, and a limitation of this study was the inclusion of only AIS. Previous studies have demonstrated significant differences in Cobb angle measurements between juvenile idiopathic scoliosis (JIS) and AIS, as well as noncongenital and congenital scoliosis (8). Specifically in JIS, end vertebrae selection was more difficult when compared with AIS. Authors noted that the vertebrae appeared with less demarcated end plates in JIS compared with AIS (15). Our study was also conducted in an urban setting, and there may be additional barriers for patients in rural setting, namely access to both primary and tertiary care, as well as access to radiographic imaging centres. Despite the limitations, this is the first study to perform a comparison of radiographic reporting of Cobb angle alongside other spinal measurements between primary and tertiary care physicians. Moreover, this study replicated a “real-life” scenario, as clinicians performed all measurements digitally using software utilised in the entire province. Previous studies have either introduced a new software or had participants perform radiographic analysis manually using digital copies.

By improving the agreement between primary and tertiary care physicians, waitlist times could be improved. One way to improve appropriate referrals to tertiary care centres may be by employing a standardised form primary providers complete as part of the referral process. These referrals could then be screened by physician assistants or nurse practitioners for completeness. In terms of agreement of Cobb angle measurement between radiologist and spine surgeons, employing a standardised dictation form for radiologists to use when reading the radiograph may help improve the accuracy measurements. This improvement could allow for timely access to patients with curves, while they are eligible for bracing treatment. Discrepancies between spinal measurements resulted in an inappropriate referral for every five referrals. Majority (57%) of these inappropriate referrals were children who did not fit the criteria for bracing treatment and required corrective spinal surgery. The remaining patients did not fit the criteria and (by improving the measurement of Cobb angle measurements in the community) these patients would be best monitored via observation by their primary care provider.

Majority of curves are often first detected by patients themselves or family members, prompting them to seek medical care from a family physician. A retrospective study conducted at Hospital for Sick Children in Toronto, Canada, amongst others, have demonstrated that most patients at initial presentation are not candidates for bracing therapy (Level 1 Evidence) (16,17). The main factor for not qualifying patients for bracing therapy was that patients had reached skeletal maturity. These findings are similar to what we report here; however, majority of our patients were not candidates for bracing treatment as their curvatures were not within the range qualifying them for bracing therapy rather than solely skeletal maturity. This highlights similar challenges of appropriate referrals to tertiary care for the management of AIS across two different centres across Canada. Findings from our study demonstrate that the removal of premature referrals may positively impact on wait times in our centre, and timely referral of patients with scoliosis curves within the range for bracing may help to avoid more invasive interventions.

## CONCLUSION

There is a disparity in spinal measurements between tertiary and primary care levels resulting in clinically significant discrepancies in referrals and triaging. Future research should explore methods to improve triaging using allied health professional as outlined above.
